# Vitamin D Status among Patients Admitted to a Geriatric Ward—Are Recommendations for Preventing Its Deficiency Effective Enough?

**DOI:** 10.3390/nu16020193

**Published:** 2024-01-06

**Authors:** Maksymilian Adam Lech, Marcin Warpechowski, Aleksandra Wojszel, Justyna Rentflejsz, Marta Świętek, Zyta Beata Wojszel

**Affiliations:** 1 Interdisciplinary Student Scientific Society, Department of Geriatrics, Medical University of Bialystok, 15-471 Bialystok, Poland; maksymilian.lech1@gmail.com (M.A.L.); warpechowski.marcin@gmail.com (M.W.); aleksandrawojszel@gmail.com (A.W.); 2 Doctoral School, Medical University of Bialystok, 15-089 Bialystok, Poland; justyna.rentflejsz@sd.umb.edu.pl; 3 Department of Geriatrics, Medical University of Bialystok, 15-471 Bialystok, Poland; 4 Department of Geriatrics and Internal Medicine, Hospital of the Ministry of Interior in Bialystok, 15-471 Bialystok, Poland; kmar@vp.pl

**Keywords:** vitamin D, vitamin D deficiency, vitamin D inadequacy, aged, frail older adults, dietary supplements, supplementation, odds ratio, logistic regression, recommendations

## Abstract

Despite a decade of available recommendations aimed at preventing vitamin D (VD) deficiency in Poland, the efficacy of these measures among community-dwelling older individuals remains inconsistent. The PolSenior2 study provided valuable insights into VD status among community-dwelling older individuals in Poland. However, it is important to note that this research did not include the elderly living in care institutions. Therefore, our study concentrates on evaluating VD status in older patients admitted to a geriatrics ward to indirectly assess their adherence to existing recommendations and preventive actions in this particular setting (whether they translate into health-promoting behaviors (i.e., taking vitamin D supplements) and whether the recommended, optimal 25(OH)D concentration values are achieved). This approach offers a comprehensive understanding of VD status in a previously understudied population. We aimed to evaluate VD status in patients aged 70 and above within the geriatrics ward, exploring its association with age, sex, BMI, and the use of VD supplements. The study involved the measurement of serum VD concentration in 240 individuals. Of these participants, 177 (73.8%) were women, and 193 (80.4%) were over 75 years old. The median 25(OH)D concentration was found to be 22.95 (IQR, 13.7–33.0) ng/mL. Notably, profound deficiency (<10 ng/mL) was noted in 15% of the participants, while 67.5% exhibited VD inadequacy (<30 ng/mL). It is worth mentioning that only 18.3% of individuals took VD supplements preadmission. Insufficiency was more prevalent in nonsupplemented individuals (70.9% vs. 52.3%, *p* = 0.02) and those with a BMI ≥30 kg/m^2^ (76.2% vs. 59.2%, *p* = 0.007). The logistic regression model demonstrated that obese patients had over two times higher odds of VD inadequacy (OR = 2.21, *p* = 0.0074), as did nonsupplemented individuals (OR = 2.23, *p* = 0.0187). The high prevalence of VD deficiency and inadequacy in geriatric ward admissions emphasizes the urgent need for targeted interventions and enhanced education for older adults, caregivers, and physicians to improve adherence to preventive supplementation practices.

## 1. Introduction

Vitamin D (VD) constitutes a group of fat-soluble steroid chemicals that play an essential role in various physiological processes. It influences and maintains the condition of the skeletal system as its primary function is related to calcium–phosphate metabolism. Deficiency of VD in older adults is associated with osteoporosis, osteomalacia, myalgia, and sarcopenia. Studies focused on the holistic understanding of the effect of VD on the human body suggest that maintaining an optimal VD level, coupled with physical activity and proper nutrition, may mitigate the risk of musculoskeletal system diseases. This, in turn, positively impacts the overall quality of life for older individuals [1]. A low concentration of 25(OH)D in the serum (an indicator of VD resources in the body) is associated with a higher incidence of cardiovascular diseases, compromised immune system functioning, depression, and neurodegenerative disorders such as Alzheimer’s disease [1,2,3,4,5]. In addition, some studies indicate that maintaining an appropriate VD level reduces the risk of developing colon, breast, ovarian, and prostate cancer [6]. However, it is crucial to note that while a meta-analysis of randomized trials showed that it had no role or effect on cancer incidence, it demonstrated a reduction in cancer-related mortality once cancer was diagnosed [7]. Despite these promising observations regarding various cancers, there is an evident need for well-designed research in this area [8]. In light of the current knowledge, it is clear that VD plays a significant role in the body, and efforts should be made to maintain its appropriate level in the serum.

**Figure 1:**
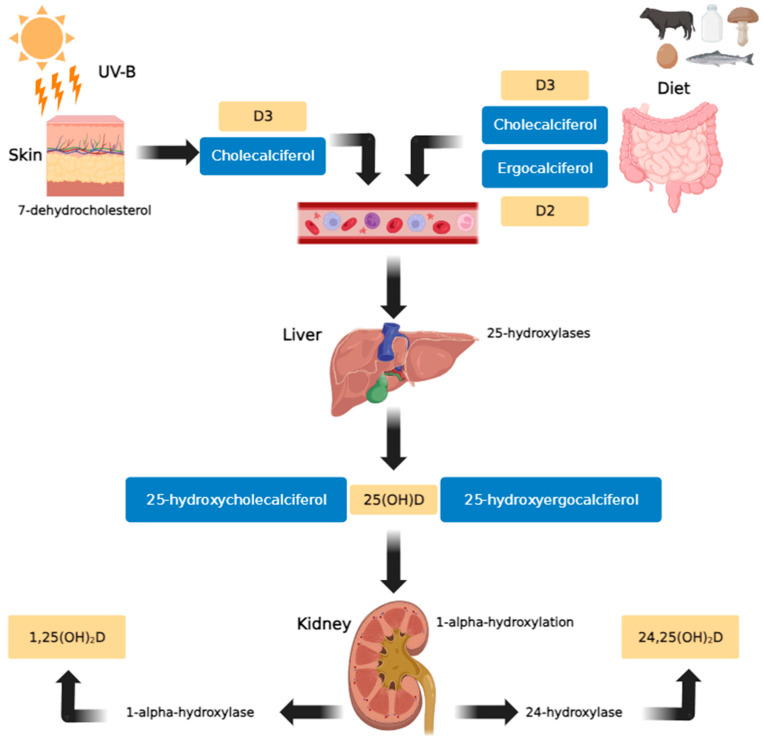
Vitamin D metabolic pathway (created using GIMP 2.10 software). In the skin, exposure to sunlight (UV-B) transforms provitamin D3 (7-dehydrocholesterol) into previtamin D3. Vitamin D3 is produced from previtamin D3 in the basal layers of the epidermis and is absorbed from natural and fortified foods and supplements (D2, D3) in the intestines. It then binds to vitamin D binding protein (DBP) in the bloodstream, before being transported to the liver and hydroxylated there by 25-hydroxylases. In the kidneys, 25-hydroxycholecalciferol [25(OH)D] (calcifediol or calcidiol) is hydroxylated by 1α-hydroxylase, resulting in the formation of the active secosteroid 1,25(OH)2D (calcitriol), which then affects various target tissues. The synthesis of 1,25(OH)2D is stimulated by parathyroid hormone and inhibited by calcium, phosphates, and 1,25(OH)2D [18].

In human plasma, VD is present in the forms of cholecalciferol (vitamin D3) or ergocalciferol (vitamin D2). Cholecalciferol is formed in the skin from 7-dehydrocholesterol under the influence of UV rays (particularly solar ultraviolet B radiation (UV-B)), and ergocalciferol is obtained as a component of plant-based foods [7]. Both variants exhibit similar biological activities. Within the liver, both cholecalciferol and ergocalciferol undergo 25-hydroxylation to form 25-hydroxycholecalciferol and 25-hydroxyergocalciferol, respectively. Subsequently, in the kidney, 1-alpha-hydroxylation occurs to produce 1,25-dihydroxyergocalciferol and the more metabolically active 1,25-dihydroxycholecalciferol (calcitriol). The enzyme 24-hydroxylase further converts 25(OH)D to 24,25(OH)2D, an inactive metabolite (Figure 1) [9].

VD participates in the regulation of calcium and phosphate metabolism, influencing bone metabolism, promoting bone mineralization by osteoblasts, and increasing the absorption of calcium and phosphates in the intestine. Its role in the pathogenesis of several diseases also results from its so-called “nonclassical,” pleiotropic effects. The presence of VD 1-hydroxylase in the cells of many tissues (known as peripheral hydroxylases) and local, peripheral production of 1,25(OH)2D have been demonstrated [9]. VD exerts its effects by activating the membrane receptor (MARRS, membrane-associated, rapid response steroid-binding) and VD receptors (VDR) present in the nuclei of most cells. These transcription factors regulate the expression of over 2000 genes responsible for cell differentiation, proliferation, and apoptosis [10]. A significant portion of the VD in the human body is produced in the skin following exposure to solar ultraviolet B radiation (UV-B) [11]. In the absence of sufficient biosynthesis in the skin, which is common among the older population, dietary intake becomes the most critical source of VD.

The primary sources of VD in the diet are the few products that naturally contain VD (e.g., fatty fish, beef, egg, egg yolk, veal, mushrooms, liver), fortified foods (cereal and dairy products), and supplements [12]. Notably, a deficiency in VD has been associated with an increased risk of cancer development [13]. Recognizing the importance of VD and a healthy diet, the World Cancer Research Fund recommends them as key elements for cancer prevention. Following these guidelines has shown a significant reduction in the risk of developing colorectal cancer, as indicated by the subjects of a recent study conducted by Serrano and co-workers [14]. According to the current recommendations, VD supplementation based on cholecalciferol for older adults is recommended throughout the year. For younger seniors (65–75 years), due to decreased efficacy of the skin synthesis, a dose of 1000–2000 IU/day (25–50 µg/day) is recommended; for older seniors (>75–89 years) and the oldest individuals (90 years and older), due to decreased efficacy of the skin synthesis, potential malabsorption and altered metabolism of VD, a higher dose of 2000–4000 IU/day (50–100 µg/day) is recommended [15,16]. When determining the quantity individually, one should account for factors such as age, body weight, VD intake from food, comorbidities, and medications taken [15]. An essential aspect when determining the dosage is obesity. Recommendations for supplementation in individuals diagnosed with obesity indicate the need to double the daily VD intake amount compared to those without obesity. Therefore, obese people over 75 should take 4000–8000 IU/day (100–200 µg/day). In cases of VD deficiency, doses should be appropriately higher, adjusted to the degree of deficiency (therapeutic doses) [15,16,17].

Despite widely available knowledge regarding the importance of VD for human health and published international recommendations aimed at preventing VD deficiency, it remains a global public health challenge affecting specific vulnerable groups, including pregnant women, children with low birth weight, and older adults. The prevalence of VD deficiency remains unknown. It depends on factors such as measurement methodology, criteria for deficiency classification, socio-economic status, and the age of the population under study [19]. Published data indicate that approximately 40% of the European population has a VD concentration (assessed as serum 25(OH)D) not exceeding 20 ng/mL (<50 nmol/l), and 40–80% of the older population struggle with VD deficiency [20]. High percentages of inadequate VD levels among seniors are found regardless of gender, skin color, or place of residence [21]. The nationwide PolSenior 2 study conducted in Poland from 2016 to 2020 showed that VD deficiency (25(OH)D concentration <20 ng/mL) was present in half of the participants over 60. The authors of the study indicated the need to consider VD supplementation when establishing the fundamental canon of public health activities for seniors in Poland [22,23,24]. Thanks to the PolSenior2 study, we now have up-to-date and relatively comprehensive information on VD status in Polish community-dwelling older people. However, the research did not include the elderly living in care institutions, or those who are hospitalized, more disabled, or diseased. Therefore, our study aims to assess VD concentrations in older patients admitted to a geriatric ward and to evaluate the adherence to existing recommendations and preventive actions in this particular setting (whether they translate into health-promoting behaviors (i.e., taking vitamin D supplements) and whether the recommended, optimal 25 (OH) D concentration values are achieved). This assessment may offer insights into the effectiveness of current recommendations within this specific group, indicating potential areas for additional actions in addressing VD deficiency.

## 2. Materials and Methods

This study included patients admitted to the Department of Geriatrics at the Hospital of the Ministry of Interior and Administration in Bialystok, Poland, between May 2019 and January 2020. One of the admission criteria, in alignment with the principles of the National Health Fund, was a minimum age of 60 years. However, the specific health issues of individuals referred to geriatrics departments often result in the majority being over 70 years old [25,26,27].

Each patient underwent a comprehensive geriatric assessment (CGA), which included a detailed medical history, physical examination, and laboratory tests. One of the crucial elements was evaluating the patient’s nutritional status, with a focus on the levels of essential vitamins. In the analysis, we considered socio-demographic data (age, gender, place of residence), anthropometric parameters (BMI), and VD concentration. We specifically included patients for whom 25-hydroxyvitamin D (25(OH)D, including 25(OH)D2 and 25(OH)D3) levels were determined, considering it the most reliable indicator of VD status [16]. Total vitamin D/25(OH)D concentration measurements were conducted using the Cobas Pure e402 immunochemical analyzer (manufacturer Roche). The chemiluminescence method, based on an immunochemical reaction using the biotin–streptavidin system, was employed for accurate assessment.

In addition to these measurements, information was gathered on the intake of VD supplements when patients were admitted to the hospital. These data were considered indicative of compliance with recommendations for supplementation and the prevention of VD deficiency. The recommendations in force in Poland at the time of the study stated that older adults should be constantly supplemented with vitamin D throughout the year, in a dose adapted to body weight (higher doses for obese people) and age. Seniors (65–75  Years), due to decreased efficacy of the skin synthesis, should receive a dose of 800–2000  IU/day. The eldest seniors (>75  Years), due to decreased efficacy of the skin synthesis, potential malabsorption, and altered metabolism of vitamin D, should be provided with a dose of 2000–4000 IU/day. The optimal range of vitamin D concentration, which should be strived for (25 (OH)D = 30-50 ng/mL), suboptimal concentration (>20–30  ng/mL), deficiency (>10–20  ng/mL), and severe deficiency (0–10  ng/mL) were also indicated, as well as high concentrations (>50–100  ng/mL) and toxic concentrations (>100  ng/mL) [16].

### 2.1. Study Parameters

Following the most recent guidelines for the prevention and treatment of VD deficiency in Poland [15,16,17], patients were stratified according to their 25(OH)D concentration as follows:

- Severe VD deficiency: level in the range of 0–10 ng/mL;
- VD deficiency: >10–20 ng/mL;
- Suboptimal level (insufficiency): >20–30 ng/mL;
- Optimal range: >30–50 ng/mL;
- High level: >50–100 ng/mL;
- Toxic range: >100 ng/mL.

For the current analysis, a concentration of 25(OH)D <30 ng/mL was treated as VD inadequacy because a concentration of VD >30 ng/mL is regarded as the optimal level one should aim for to achieve optimal benefits from VD status.

BMI [kg/m^2^] was stratified according to World Health Organization guidelines [28] as follows:

- <18.5—underweight;
- 18.5–24.99—normal weight;
- 25.0–29.99—overweight;
- 30 and over—obesity.

### 2.2. Statistical Analysis

The data collected for analysis were processed using the IBM SPSS Statistica Version 27 Software suite (SPSS, Chicago, IL, USA) and R version 4.3.1. The Kolmogorov–Smirnov test was used to assess the distribution of variables. Data were presented as mean (M) and standard deviation (SD) for normally distributed continuous variables, median (Me) and interquartile range (IQR) for non-normally distributed ones, and the number of cases and percentages for categorical variables. Proportions were compared using χ^2^ tests or Fisher’s exact test, as appropriate, while the independent samples Student’s *t*-test and the Mann–Whitney U-test were used to compare the distribution of continuous variables. For comparisons among more than two groups, Kruskal–Wallis and post hoc tests were used. Logistic regression (direct method) was used to create a multivariable model to assess the association between VD inadequacy and selected predictors. Odds ratios with a 95% confidence interval (CI) and *p*-value were estimated. Only VD deficiency predictors with a *p*-value lower than 0.1 were included in the model. The cut-off point was set at 0.5, and each model underwent ten-fold cross-validation. Missing values were omitted, and statistics in such cases were calculated for the adequately reduced groups. A two-tailed *p*-value of less than 0.05 was considered statistically significant in all analyses.

### 2.3. Ethics Approval

The study was approved by the Ethics Committee at the Medical University of Bialystok (no R-I-002/326/2017). All procedures performed adhered to the ethical standards set by the Medical University of Bialystok Ethics Committee in accordance with the Helsinki Declaration and its subsequent amendments. This study adheres to the usual practice protocols. Informed consent for participation was obtained from study participants or their guardians.

## 3. Results

The study included 240 out of 250 patients consecutively admitted to the geriatric ward, with 177 (73.8%) being women and 193 (80.4%) aged 75 and above. The average age for men was 81 (±7.65) years, and for women, it was 80 (±6.27) years, with no statistically significant difference between the genders. The median 25(OH)D concentration for the entire study group was 22.95 (IQR 13.7–33.0) ng/mL. Breaking down by gender, the median VD concentration for women was 23 (IQR 14.0–34.0) ng/mL, and for men, it was 22 (IQR 13.0–32.0) ng/mL, with no significant differences observed in VD concentration between the sexes (Table 1). In terms of age groups, patients aged 60–74 showed a median VD concentration of 24 (IQR 14.0–33.0) ng/mL, while those aged 75 and above had a median VD concentration of 19.8 (IQR 13.0–30.0) ng/mL (*p* > 0.05).

**Figure 2:**
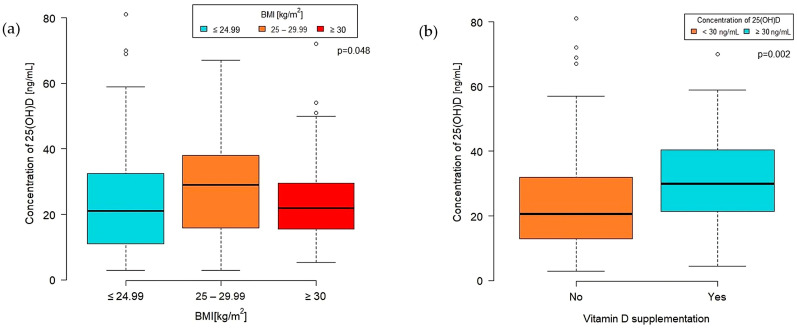
Concentration of VD by BMI group (**a**) and VD supplementation (**b**).

A significant difference in VD concentration was observed among BMI categories (*p* = 0.048) (Figure 2a). The highest median VD concentration was found in the overweight group (BMI 25–29.99 kg/m^2^) at 29 (IQR 16.0–38.0) [ng/mL]. Patients classified as obese (BMI 30 kg/m^2^ or more) had a median VD concentration of 22 (IQR 15.00–29.60), while those with a normal BMI level (<25 kg/m^2^) had a median of 21 (IQR 11.00–32.50) [ng/mL].

**Figure 3:**
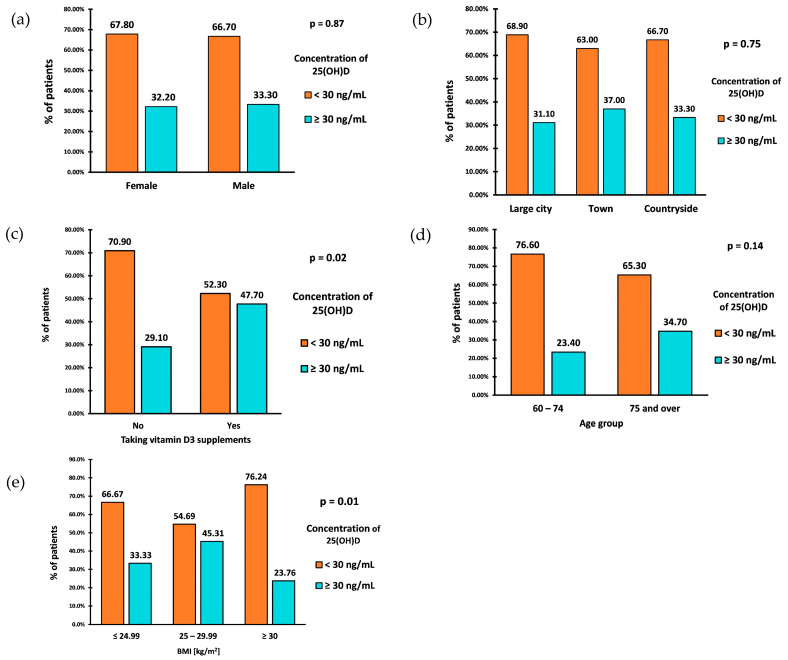
Prevalence of VD inadequacy by sex (**a**), place of residence (**b**), intake of vitamin D supplements (**c**), age (**d**), and BMI (**e**).

The prevalence of VD inadequacy (25(OH)D < 30 ng/mL) in the study group was 67.5% (162 patients). The deficiency in 15% of cases was profound, with a VD level of <10 ng/mL. The percentage of VD inadequacy was similar between the sexes (Figure 3a). Individuals aged 60–74 had a slightly higher prevalence of VD inadequacy than those aged 75 and older (Figure 3d). However, there were no statistically significant differences in VD inadequacy between sex and age groups.

The prevalence of VD inadequacy in the study group was also not associated with the place of residence (*p* = 0.753) (Figure 3b). However, those living in the countryside exhibited the highest average VD concentrations, with a median VD concentration of (IQR: 17.00–33.60). Similar median values of VD concentrations were observed among those living in large cities (Me: 22; IQR: 13.00–33.00) and towns (Me: 22.50; IQR: 15.00–37.00).

A parameter significantly associated with the prevalence of VD inadequacy was BMI (*p* = 0.01). Patients classified as overweight (BMI 25–29.99 kg/m^2^) had the lowest prevalence of VD inadequacy, with a rate of 54.69% (Figure 3c). The prevalence of inadequacy among patients with a normal BMI (18.5–24.99 kg/m^2^) was 66.67%. Patients diagnosed with obesity (BMI ≥ 30 kg/m^2^) showed the highest prevalence of VD inadequacy, equal to 76.24%. Calculating OR of VD inadequacy between patients diagnosed with obesity (BMI ≥ 30 kg/m^2^) and those without obesity (BMI < 30 kg/m^2^) resulted in OR = 2.21 (95% CI: 1.24–3.95) (*p* = 0.0074). This indicates that the odds of VD inadequacy are 2.21 times higher in patients diagnosed with obesity than in the group without obesity.

Furthermore, the concentration of 25-hydroxyvitamin D was significantly higher in the group taking VD supplements than those who did not (*p* = 0.002). The median VD concentration in the supplementation group was 29.80 (IQR 21.50–40.50) ng/mL, whereas in the nonsupplementation group, it was 20.50 (IQR 13.00–32.00) ng/mL (Figure 2b).

Only 44 patients (18.33%) reported taking VD supplements. Among the 196 (81.67%) who did not take supplements, 139 (70.9%) were found to have VD inadequacy, while 57 (29.10%) had proper levels (Figure 3c). Among those who claimed to take supplements, 23 (52.30%) had VD inadequacy, while 21 (47.70%) had proper levels. The statistical analysis demonstrated a significant association between taking VD supplements and experiencing inadequacy (*p* = 0.017). The odds of VD inadequacy in the nonsupplemented VD group were 2.43, while the odds of inadequacy in the supplemented group were 1.09. After calculating the odds ratio, the result was 2.23 (95% CI: 1.14–4.34) (*p* = 0.0187). This indicates that the odds of VD inadequacy in the nonsupplementing VD group are 2.23 times higher than in the supplementing VD group. We analyzed the relationship between the occurrence of insufficiency of vitamin D concentration (i.e., <30 ng/mL) and sex, age, place of residence, and BMI separately for the groups taking and not taking vitamin D supplements. The results were similar and presented together. There was no significant association between age, gender, and the use of VD supplements, respectively (Table 2). There was also no significant association between the place of residence and the use of VD supplements.

In the multivariable logistic regression assessing the concurrent association between VD inadequacy, supplementation, and obesity diagnosis, the AUC was 0.625 (±0.03) (Table 3). It was found that by holding the obesity diagnosis constant, the odds of VD inadequacy increased by 128% (95% CI: 1.13–4.58) for patients not supplementing VD compared to supplementing VD. It was also found that by holding VD supplementing constant, the odds of VD inadequacy increased by 112% (95% CI: 1.19–3.86) for obese patients compared to nonobese patients.

## 4. Discussion

Our research confirmed that in older patients admitted to the Department of Geriatrics, VD concentration is below the appropriate value in most cases. The concentration of 25(OH)D ranged from 3 to 81 ng/mL, but it was within the optimal level (i.e., within the range ≥ 30–50 ng/mL) in only 25% of patients (Table 1). Only 6.7% had a high level, i.e., ≥50 ng/mL, whereas potentially toxic levels, i.e., >100 ng/mL, were not found. Therefore, as our study shows, most older patients of the geriatrics ward, regardless of age, sex, or place of residence, are inadequate in VD. Older people are at risk of VD insufficiency due to age-related impaired skin biosynthesis, more frequently observed inadequate absorption and metabolism [1]. According to some studies, nursing home residents and older inpatients are at the highest risk for VD deficiency [29].

In the nationwide PolSenior2 study in 2018–2019 among community-dwelling older people in Poland, severe VD deficiency was found in 11.5% of respondents, deficiency in 44.8%, and a suboptimal level in 30.9%. Only 12.7% of the subjects had a concentration of VD within the optimal range, defined as ≥30 ng/mL [22]. Compared to these results, the VD status of geriatrics ward patients participating in our study in 2019 was better, probably due to their frequent use of medical advice, including the geriatrics outpatient clinic, and the opportunities to receive recommendations on proper vitamin D3 supplementation.

A study conducted in 2019 in Europe and the Middle East indicates that low VD levels may affect up to 40% of the general population with 12.5% experiencing a severe deficiency [21]. Seniors and nursing home residents had worse results than the general population. It is also worth emphasizing that the percentage of people with a deficiency is relatively low in Scandinavian countries despite unfavorable geographical conditions (due to the geographical latitude, skin synthesis is significantly limited) [17]. Between 6.6% and 33.6% of the population of northern Europe has VD < 20 ng/mL, while in Western Europe, this prevalence range varies from 27.2% to even 61.4%. The most crucial factor here is widely used preventive measures such as VD supplementation, consumption of products rich in it (e.g., cod liver oil), and fortifying food with this vitamin. The estimated supply of VD in northern countries is about 10 mcg/day on average, while in Western European countries, it ranges from 1.5 mcg/day to about 5 mcg/day [21]. Unfortunately, there is no standardized data on this subject for Eastern Europe. Still, the deficiency affects a more significant percentage of the population than in Northern or Western European countries, and the average concentration is usually below 20 ng/mL.

In cross-sectional studies, the lowest average value was observed in Germany (39.50 ± 7.50 nmol/L) (15.82 ± 3.00 ng/mL), while the highest average concentrations were observed in Austria (56.50 ± 30.70 nmol/L) (22.63 ± 12.29 ng/mL) and Spain (50.70 ± 29.2 nmol/L) (20.31 ± 11.69 ng/mL) [25,26]. The incidence of VD deficiency is estimated from 30% of the elderly population in Germany to as high as 60% among Eastern European countries [21,30,31]. Retrospective research from the United Kingdom, analyzing data from 2005 to 2015, states that approximately 30% of surveyed women and men over the age of 65 years have a confirmed deficiency [32].

Therefore, despite the commonly available knowledge on the importance of VD for human health and published international recommendations on preventing its deficits, VD deficiency is a global public health concern. In 2009, the Polish standardized guidelines for VD supplementation were established [33]. The recommended supplementation dose for adults ranged between 800 and 1000 IU/day, with no specific recommendations for individuals aged over 75 years. In 2013, new guidelines for the Central European population were published, introducing many changes to the reference values. The proposed optimal concentration was set between 30 and 50 ng/mL with a more stringent deficiency threshold of 20 ng/mL [34]. The adult supplementation dose range has been increased, setting it at 800–2000 IU/day. However, there were still no specific recommendations for seniors over 75 years. The subsequent change occurred in 2018, with the next update of recommendations [16]. It suggested that older seniors should use a higher dose, ranging between 2000 and 4000 IU/day, depending on their weight and initial VD concentration in plasma. It also distinguished a deficiency in the range of 10–20 ng/mL, and values below 10 ng/mL were classified as a severe deficiency. The latest guidelines from 2023 still maintain the previously established reference values, only raising the lower dose limit for supplementation to 1000 IU/day instead of 800 IU/day [15]. Nevertheless, despite the prevention standards having been in force for many years, instances of severe VD deficiency persist in this population. Our research has shown that it is essential to consider a higher supplementation dose for obese individuals. The presence of obesity more than doubles the likelihood of an inadequate VD concentration, even with controlled supplementation. The reason for the increased need for VD augmentation in people with obesity is not immediately apparent. Increased VD clearance or impaired VD synthesis does not seem to be the correct explanation, as the values do not differ between the obese population and that of normal-weight individuals. Therefore, at this point, the most relevant factor seems to be excessive “dilution” due to increased mass and tissue volume in obese individuals [35].

The use of vitamin, mineral, and fish oil supplements is common among older people living in the community outside of care institutions, and studies suggest an increase in their consumption, particularly of calcium and VD, over the years [36]. According to current guidelines, VD supplementation in the senior population should be maintained year-round [15,36]. Despite the fact that recommendations for preventive VD supplementation have been in force in Poland for about 15 years, the prevalence of inadequacy and deficiency remains high, suggesting that the number of seniors adhering to these recommendations is meager. In the nationwide PolSenior2 study conducted in 2018–2019, only 16.3% of women and 6.4% of men over 60 reported taking vitamin D3 supplements [22]. Consequently, in both groups—the general population of seniors in the PolSenior2 study and those hospitalized in our study—most elderly subjects had VD inadequacy.

Based on the available literature, improving the VD status among older adults will require a multifaceted approach. One crucial step is to intensify the focus on educating the community, particularly seniors. The potential challenge lies in the low compliance observed among older patients, especially when they are physically and cognitively impaired. It then becomes imperative to educate their caregivers properly. This issue has been identified in care institutions, and adequate education and training of staff responsible for nursing home residents has demonstrated improvement in addressing VD deficiencies [37].

Research conducted in Poland highlights the importance of extending educational efforts to include physicians, particularly family physicians, as they are the first line of contact for patients. The study titled “Vitamin D Knowledge, Attitudes, and Practices of Polish Medical Doctors” analyzed doctors’ knowledge of VD in the context of their clinical practice and personal application of the recommendations. The study involved 701 medical doctors from various specialties, predominantly women, working in diverse settings. The findings revealed that approximately 25% of the participants had their VD levels assessed, and 62% were found to have low levels. Although 82% reported that they provide information to patients about recommendations regarding supplementation, only around 40% did so for most patients. Alarmingly, 18% of surveyed doctors never discussed recommendations concerning VD supplementation with their patients [38]. This is crucial because both the study conducted in the geriatric ward and the nationwide PolSenior2 study demonstrate that VD supplementation significantly improved VD status. In both studies, a majority of seniors receiving VD preparations achieved optimal levels of 25(OH)D.

According to some authors, maintaining an optimal VD concentration requires a combination of education, supplementation, and regular monitoring of VD levels [39]. Boluses of higher doses of VD3 may improve compliance with prophylactic supplementation and deficiency therapy recommendations. The extended serum half-life (4–6 weeks) and tissue storage of the circulating metabolite of VD allow for less frequent administration, such as every other day, twice a week, or once a week. Intervention studies indicate that preparations containing higher VD doses can help achieve and maintain the appropriate, stable level of 25(OH)D. However, if the level remains insufficient, patients should avoid excessively escalating VD doses in boluses. Instead, in such cases, the intervals between doses should be shortened [40].

Another potential measure can be a more significant enrichment of commonly consumed food products like margarine, milk, or eggs with VD [41,42]. As the modeling results for Canada’s population suggest, while this policy could improve VD levels for individuals not taking VD supplements, the overwhelming majority of Canadians (76%) would still fall below the estimated average requirement (10 mg/day or 400 IU/day). Therefore, it remains crucial to promote VD supplementation to address population-level VD inadequacies [43,44].

The high quality of VD supplements is also very important [45]. In the context of Poland, it is important to note that only a few of the VD preparations available in the market have the status of a “drug,” ensuring a reliably assessed content of cholecalciferol in each tablet. Some older people may take various multivitamins with differing VD content and possibly varying bioavailability, which could be a contributing factor [46]. Efforts should be made to ensure that the VD supplements consumed by older people are of high quality.

When interpreting the results of the presented study, it is important to consider its limitations, which constrain the extent of the conclusions that can be drawn. This is a single-centered study concentrating on VD intake upon admission to the department, with no inquiry into past or periodic supplementation. Information about the type of supplement (whether VD or multivitamin preparations) and the dosage taken by patients is not available. This study neither assesses the patient’s/caregiver’s knowledge on this topic nor the reasons for supplement intake. Additionally, the analysis did not include questions about sun exposure and consumption of VD-enriched foods. It is important to perceive this analysis as a preliminary study, indicating the need for further investigation.

## 5. Conclusions

The conducted study showed that despite recommendations for VD supplementation having been in place for over a decade, the percentage of seniors admitted to the Department of Geriatrics with VD deficiency and inadequacy remains alarmingly high. This emphasizes the urgent need for further actions to address this concerning trend. These actions should include educational initiatives targeting older adults and their caregivers, along with enhancing the knowledge of physicians caring for seniors. An assessment of activities promoting VD supplementation should be conducted to optimize their effectiveness. Additionally, research on available VD supplements in the market is crucial, considering that only select preparations in Poland hold “drug” status and meet quality standards. Modifications of therapy methods, using preparations containing higher doses of VD, may help to improve compliance by older adults. Improvement at the population level may also be brought about by promoting the availability of food products fortified with VD or increasing the degree of this food enrichment.

## Figures and Tables

**Table 1:** Vitamin D concentration strata in the sex groups.

25(OH)D Concentration [ng/mL]	Women (*n* = 177)		Men (*n* = 63)		Total (*n* = 240)		*p*-Value
	n	%	*n*	%	*n*	%	
<0; 10	30	16.9	6	9.5	36	15.0	0.73
<10; 20	50	28.2	22	34.9	72	30.0	
<20; 30	40	22.6	14	22.2	54	22.5	
<30; 50	43	24.3	19	30.2	62	25.8	
<50; 100	14	7.9	2	3.2	16	6.7	

**Table 2:** Use of vitamin D3 supplements by sex and age.

	Taking Vitamin D3 Supplements		*p*-Value
	Yes	No	
	*n* (%)	*n* (%)	
Sex			
Woman (*n* = 177)	33(18.6)	144(81.4)	0.13
Man (*n* = 63)	11 (17.5)	52 (82.5)	
Age			
60–74 years (*n* = 47)	5 (10.6)	42 (89.4)	0.84
75+ years (*n* = 193)	39(20.2)	154 (79.8)	

**Table 3:** Multivariate logistic regression analysis—association of vitamin D inadequacy with VD supplementation and obesity.

Coefficients	Estimate	Std. Error	Odds Ratios	95% CI	*p*-Value
(Intercept)	−1.309	0.2462	0.27	0.16–0.43	<0.001
VD supplementation (no)	0.8228	0.3542	2.28	1.13–4.58	0.02
Obesity (BMI ≥ 30 kg/m^2^)	0.7527	0.2999	2.12	1.19–3.86	0.01

## Data Availability

The data presented in this study are available on request from the corresponding author. The data are not publicly available due to privacy and ethical considerations.

## Abbreviations

VD—Vitamin D; MARRS—membrane-associated rapid response steroid; VDR—Vitamin D receptors; UV-B—ultraviolet B radiation; DBP—vitamin D binding protein; CGA—comprehensive geriatric assessment; BMI—Body Mass Index; M—mean; SD—standard deviation; Me—median; IQR—interquartile range; OR—odds ratio; CI—confidence interval.
